# Clinical Efficacy of PD-1 Inhibitors Plus Split-Course Radiotherapy in the First-Line Treatment of Advanced Kidney Cancer: A Randomized Controlled Trial

**DOI:** 10.1155/2022/8100323

**Published:** 2022-07-30

**Authors:** Weiping Li, Zhigang Cao, Pengcheng Chang, Bin Zhang, Fudong Li, Dehui Chang

**Affiliations:** ^1^Department of Urology, The First Hospital of Lanzhou University, Lanzhou 730030, China; ^2^Department of Urology, The 940 Hospital of Joint Logistics Support Force of Chinese PLA, Lanzhou 730050, China

## Abstract

**Objective:**

To assess the clinical efficacy of programmed death 1 (PD-1) inhibitors plus split-course radiotherapy in the first-line treatment of advanced kidney cancer.

**Methods:**

In this prospective, randomized, single-blinded, controlled trial, 44 patients with advanced kidney cancer initially treated in our hospital from January 2017 to December 2018 were recruited. They were concurrently and randomly assigned at a ratio of 1 : 1 to the control group and the study group, with 22 cases in each group. The control group received PD-1 inhibitor nivolumab combined with ipilimumab, and the study group received split-course radiotherapy plus. The primary endpoint is clinical efficacy, and the secondary endpoints are progression-free survival (PFS), overall survival (OS), and adverse events (AEs).

**Results:**

Nivolumab plus split-course radiotherapy was associated with an objective remission rate (ORR) of 59.09% versus nivolumab alone with an ORR of 27.27%. The median PFS was 21.5 months (95% CI: 14.1—NA) after single nivolumab therapy and 28.1 months (95% CI: 24.5—NA) after nivolumab plus split-course radiotherapy, with an HR of 1.875 (95% CI: 0.877–4.011). The median OS was 27.1 months (95% CI: 20.7—NA) after single nivolumab therapy and not reached after nivolumab plus split-course radiotherapy and an HR of 2.56 (95% CI: 1.081–6.06). Nivolumab was associated with significantly better OS plus split-course radiotherapy versus nivolumab alone.

**Conclusion:**

Nivolumab plus split-course radiotherapy in patients with advanced renal cell carcinoma significantly improves ORR and prolongs overall survival with a good safety profile.

## 1. Introduction

Kidney cancer, also known as Renal Cell Carcinoma (RCC), is a malignant tumor originating from the urinary tubular epithelial system of the renal parenchyma. Approximately 85% of renal cell carcinomas are adenocarcinomas of proximal tubular origin, followed by metastatic cell carcinoma of the renal pelvis. Surgical treatment is available for patients with early-stage renal cell carcinoma without metastasis or locally progressive renal cell carcinoma, but patients with advanced metastatic renal carcinoma are inoperable and require comprehensive medical management [1, 2]. Due to the insensitivity of renal cell carcinoma to radiotherapy and chemotherapy, cytokine therapy such as interleukin and interferon has been mainly performed since the 1990s, but the objective response rate (ORR) only ranges from 5% to 27%, and the median progression-free survival is only 3–5 months with significant adverse events [3, 4]. About 17% of patients have already developed advanced kidney cancer at the time of diagnosis, and about 50% of initially treated patients eventually progress to an advanced stage [5]. There exists an urgent need to explore new therapeutic approaches to improve the prognosis of patients with advanced kidney cancer.

PD-1 inhibitors provide new therapeutic approaches for patients with advanced cancer [6], and the PD-1 inhibitor pembrolizumab is an approved drug for the treatment of malignancies such as melanoma, non-small cell lung cancer, head and neck squamous cell carcinoma, classical Hodgkin's lymphoma, uroepithelial carcinoma, and cancers with high microsatellite instability [7, 8]. PD-1 inhibitors feature the advantages of broad-spectrum, low toxicity, durability, and broader anticancer effects based on biomarkers rather than tumor sources. PD-1 inhibitors, if effective, may allow prolonged survival or even clinical cure of patients with advanced tumors and are less liable to develop drug resistance. Unlike conventional radiotherapy, the principle of immunotherapy with PD-1 inhibitors is to activate the immune system for tumor attack instead of enhancing the killing effect of the drug, resulting in much fewer overall side effects [9, 10]. In addition, PD-1 inhibitors play an essential role in tumor immune escape by enhancing the function of CD8+ T cells and CD4+ Th cells and inhibiting the development of various tumors [11].

Split-course radiotherapy is a radiotherapy splitting modality with a single dose greater than the conventional dose, a lower total radiotherapy dose, and a smaller number of radiotherapy sessions versus conventional splitting. It shortens the duration of the treatment without reducing the total relative biomass, with potentially improved efficacy. Split-course radiotherapy is generally delivered at high doses of 3–5 Gy per day or even higher, with a few irradiations completed in 1 to 2 weeks [12]. Stereotactic split-course radiotherapy is a combination of stereotactic radiotherapy and split-course radiotherapy and has become an alternative to single stereotactic radiotherapy and conventional split-course radiotherapy [13]. It has been shown that radiotherapy, especially stereotactic split-course radiotherapy, releases a large number of tumor antigens from the irradiated lesion, which promotes antigen presentation by DCs cells, activates CD8+ T cells, CD4+ T cells, and NK cells stimulates the immune response, and regulates the changes in the tumor and its microenvironment, resulting in an “in situ vaccine” effect [14].

Radiotherapy modulates tumor phenotype, enhances antigen presentation and tumor immunogenicity, increases cytokine production, and alters the tumor microenvironment, thereby killing tumors by enhancing the immune system [15]. Immunotherapy promotes host anti-tumor immune response and its combination with radiotherapy results in a more efficient anti-tumor response versus stand-alone radiation therapy or immunotherapy. Meta-analyses have shown that combination therapy with PD-1/PD-L1 inhibitors and radiotherapy may improve progression-free survival (PFS), overall survival (OS), and ORR in patients with advanced NSCLC without increasing serious adverse events [16]. However, PD-1 inhibitors plus radiotherapy in kidney cancer are marginally explored. The present study achieved promising results in patients with advanced kidney cancer treated with PD-1 inhibitor nivolumab/ipilimumab plus stereotactic split-course radiotherapy. The results are reported below.

## 2. Materials and Methods

### 2.1. Research Design

In this prospective, randomized, single-blinded, controlled trial, 44 patients with advanced kidney cancer initially treated in our hospital from January 2017 to December 2018 were recruited. They were concurrently and randomly assigned at a ratio of 1 : 1 to either a control group or a study group, with 22 cases in each group. The study was approved by the ethics committee of the First Hospital of Lanzhou University (approved no. 2017-C22/334) and all patients provided written informed consent as per the Declaration of Helsinki principles.

### 2.2. Inclusion and Exclusion Criteria

#### 2.2.1. Inclusion Criteria

(1) Patients aged 18–80 years; (2) with histologically or pathologically confirmed metastatic RCC; (3) oligometastases (1–5 metastases) with Response Evaluation Criteria In Solid Tumors (RECIST) version 1.1 measurable lesions [17]; (4) with at least 1 metastasis eligible for split-course chemotherapy; (5) with first systemic therapy; (6) Eastern Cooperative Oncology Group (ECOG) score ≤2 points; and (7) with an expected survival of ≥12 months.

#### 2.2.2. Exclusion Criteria

(1) Patients with previous (within 4 weeks) monoclonal antibodies, targeted small molecule chemotherapy, immunosuppressants, or high-dose radiotherapy (bioequivalent dose >30 Gy); (2) with untreated or progressive intracranial metastases; (3) with malignant pleural effusion; (4) with evidence of spinal cord compression or bone injury requiring surgical fixation; (5) with active autoimmune disease or HIV infection; (6) in lactation or pregnancy; and (7) with psychoneurological disorders that prevent cooperation with treatment or adherence to follow-up.

### 2.3. Treatment Methods

Control group: induction ipilimumab (Bristol-Myers Squibb, SJ20210020, China) 1 mg/kg combined with nivolumab (GlpBio, BMS-936558, USA), 3 mg/kg every 3 weeks for cycles 1–4 followed by maintenance treatment with nivolumab 240 mg every 2 weeks or 480 mg every 4 weeks until disease progression (as determined by RECIST 1.1), intolerance, or patient/physician decision to stop treatment.

**Figure 1:**
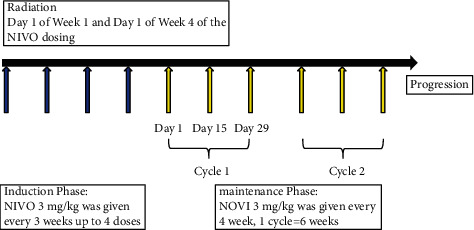
Treatment schedule.

Study group: the study group received stereotactic split-course radiotherapy plus the specific methods were as follows: patients were fixed in a supine position with a negative pressure vacuum body film device, and all lesions were scanned and localized continuously using a CT simulation and localization machine, with 4-dimensional CT scans for lung metastases, and MRI scans for brain and vertebral metastases and a treatment planning system to outline the gross tumor volume (GTV), organs at risk, clinical target volume (CTV), and planned target volume (PTV) layer by layer. The outline area was modified according to the location of the patient's metastases to assess the optimal treatment regimen. Isocentric irradiation with 6 MeV linear gas pedal radiation at a prescribed radiotherapy dose of 50 Gy/5 F was performed on the first day of the first and fourth week. Immunotherapy was administered on the day of radiotherapy and repeated on the scheduled dates. The immunotherapy and radiotherapy schedule is shown in Figure 1.

### 2.4. Endpoints

#### 2.4.1. Primary Endpoint

The primary endpoint was clinical efficacy. All patients were treated for 6 months to assess clinical efficacy, which was classified as complete remission (CR), partial remission (PR), stable disease (SD), and progressive disease (PD) per RECIST 1.1. CR: Clinical and radiological evidence of all target lesions disappeared and tumor marker levels returned to a normal scope; PR: Total longest diameter (LD) of all target lesions decreased ≥30% from baseline and no new lesions were found; PD: Total LD increased ≥20% based on the smallest target lesion since the start of treatment or the appearance of one or more new lesions or the appearance of new lesions; SD: Between PR and PD. ORR = (CR + PR)/total number of cases × 100%.

#### 2.4.2. Secondary Endpoints

Secondary endpoints for this study include PFS, OS, and AEs. All subjects were assessed for treatment efficacy every 6 weeks until treatment discontinuation, disease progression, or death. Progression-free OS was defined as patient death from any cause from the start of treatment to the time of assessment, and PFS was defined as distant metastases from the start of treatment to the time of assessment. The occurrence of adverse reactions during treatment was recorded.

### 2.5. Statistical Analyses

The SPSS 23.0 was used for data collation and statistical analyses, and the *R* language SURVIVAL package was used to plot the graphics in the study. The measurement data were expressed as $\bar{x}\pm s$ and processed using the *t*-test for intergroup comparison. The count data were expressed as rates (%) and processed by the chi-square test for intergroup comparison. Survival data were calculated using the Kaplan–Meier method to calculate the survival rate, median survival, and survival curves to describe the survival process. Differences were considered statistically significant with *α* = 0.05 as the threshold of significance.

## 3. Results

### 3.1. Baseline Data

In the control group, there were 12 maless and 10 females with an average age of 62.34 ± 15.67 years. In the study group, there were 9 maless and 13 females with an average age of 60.94 ± 13.67 years. The baseline characteristics, including age, gender, KPS scores, evaluable disease sites, histology, brain metastasis, and ECOG score of the two groups were comparable (*P* > 0.05) (Table 1).

### 3.2. Clinical Efficacy

The control group had 2 cases of PR, 4 cases of PR, 10 cases of SD, and 4 cases of PD with an ORR of 27.27% (6/22) and the study group had 6 cases of PR, 7 cases of PR, 7 cases of SD, and 2 cases of PD with an ORR of 59.09% (13/22). Nivolumab plus split-course radiotherapy was associated with a higher ORR versus nivolumab alone (*P* < 0.05) (Table 2).

### 3.3. PFS and OS

**Figure 2:**
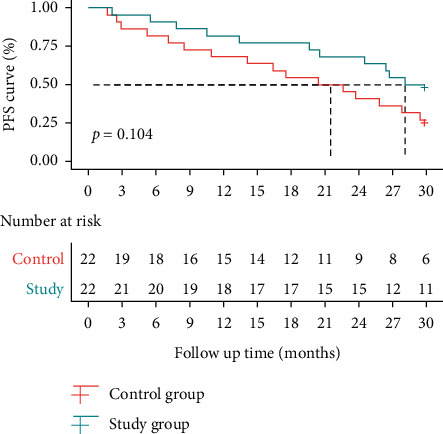
Survival curves of PFS.

**Figure 3:**
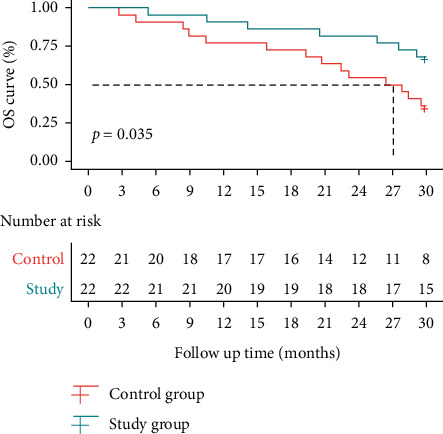
Survival curves of OS.

The median PFS was 21.5 months (95% CI: 14.1—NA) after single nivolumab therapy and 28.1 months (95% CI: 24.5—NA) after nivolumab plus split-course radiotherapy, with an HR of 1.875 (95% CI: 0.877–4.011) (*P*=0.104) (Table 3 & Figure 2). The median OS was 27.1 months (95% CI: 20.7—NA) after single nivolumab therapy and not reached after nivolumab plus split-course radiotherapy and an HR of 2.56 (95% CI: 1.081–6.06) (*P*=0.035) (Table 4 & Figure 3).

### 3.4. Adverse Events

The control group had 18 cases with adverse events and 7 cases with an adverse events grade ≥3, and the study group had 20 cases with adverse events and 12 cases with an adverse events grade ≥3. The two groups showed a similar incidence of adverse events (*P* > 0.05) (Table 5).

## 4. Discussion

In this study, the control group was treated with nivolumab combined with ipilimumab, and the study group was treated with stereotactic split-course chemotherapy plus. The results showed that the study group was associated with an objective remission rate (ORR) of 59.09% versus the control group with an ORR of 27.27% (*P* < 0.05). The median PFS was 21.5 months (95% CI: 14.1—NA) after nivolumab combined with ipilimumab and 28.1 months (95% CI: 24.5—NA) after split-course radiotherapy plus, with an HR of 1.875 (95% CI: 0.877–4.011). The median OS was 27.1 months (95% CI: 20.7—NA) after nivolumab combined with ipilimumab and not reached after split-course radiotherapy plus an HR of 2.56 (95% CI: 1.081–6.06). The study group was associated with significantly better OS plus split-course radiotherapy than the control group.

Nephrectomy or partial nephrectomy is the standard of treatment for early-stage RCC. Advanced kidney cancer is usually unresponsive to standard chemotherapy, so radiotherapy and immunotherapy are considered the primary treatment options [18]. Immune checkpoint inhibitor drugs such as pembrolizumab and nivolumab, currently used for advanced kidney cancer treatment, can enhance the immune response of antirenal cancer cells by blocking PD-1. The efficacy of PD-1 inhibitors in kidney cancer is well established. The CheckMate 025 study included 821 patients with advanced renal clear cell carcinoma who had received prior first- or second-line antiangiogenic therapy and were randomized 1 : 1 to receive either intravenous nivolumab or oral everolimus 10 mg/d, respectively, resulting in a median OS of 25.0 and 19.6 months (HR = 0.73, *P*=0.002) and the ORRs of 25% and 5% (OR = 5.98, *P* < 0.001). The incidence of grade 3–4 adverse reactions in both groups was 19% and 37%, confirming the superior efficacy and better safety profile of nivolumab over everolimus in the treatment of advanced kidney cancer [19]. The CheckMate 214 study evaluated the efficacy and safety of nivolumab plus a low-dose ipilimumab regimen versus standard first-line therapy sunitinib for first-line treatment, and the results showed significantly prolonged OS and improved ORR by nivolumab [20].

Stereotactic split-course radiotherapy is a new technique in radiotherapy that has been used for the treatment of lung, liver, and spinal malignancies, but its use in kidney cancer is mostly overlooked. Radiotherapy kills tumors and activates the host immune system, modulates tumor phenotype, enhances antigen presentation and tumor immunogenicity, increases cytokine production, and alters the tumor microenvironment, thereby enhancing the immune system to kill tumors. The combination of the two can lead to a more effective antitumor response versus radiation or immunotherapy alone. Stereotactic split-course radiotherapy plus immunotherapy regimens, mainly for oligometastatic tumors, aim to maximize the combined therapeutic efficacy of the radiation field by exploiting the “in situ vaccine” effect of radiotherapy, i.e., activation of antigen-presenting cells and deregulation of T-cell suppressive signals. Radiotherapy alters the differentiation and function of T cells and promotes the expression of PD-L1, resulting in an enhanced effect of anti-PD-L1 therapy [21]. In a study of patients with stage II unresectable non-small cell lung cancer, the addition of the PD-L1 inhibitor durvalumab after 42 days of concurrent radiotherapy was associated with progression-free survival and overall survival benefits of patients after immunotherapy. Despite the increased probability of pneumonia associated with radiotherapy, the overall safety profile is considered satisfactory [22]. In addition, research RTOG3505 investigated the efficacy of nivolumab-synchronized radiotherapy in nonsmall cell lung cancer, in which between 4 and 12 weeks after completion of concurrent chemoradiation, eligible patients were randomized to the PD-1 monoclonal antibody nivolumab 240 mg i.v. or placebo every 2 weeks for up to 1 year, and the results are promising [23]. In this study, split-course radiotherapy was used not only to eradicate large areas of progressive disease but also to provide antigen presentation and immune stimulation, which synergistically acted with PD-1 inhibitors to improve the response rate and complete response in patients with metastatic clear cell renal cell carcinoma. However, this study has the following limitations. The study is a single-center study with a short follow-up period, which prevents validation of long-term efficacy. In addition, examinations of the circulating immune cells and identification of PD-L1 expression were absent in this study, preventing a valid determination of the efficacy of immunotherapy-synchronized chemotherapy on the immune system.

## 5. Conclusion

Nivolumab/ipilimumab plus split-course radiotherapy in patients with advanced renal cell carcinoma significantly improves ORR and prolongs overall survival with a good safety profile, and its mechanism requires further investigation.

## Figures and Tables

**Table 1:** Baseline demographics and clinical characteristics.

	Control group (*n* = 22)	Study group (*n* = 22)	t/*χ*^2^	*P*
Age ($\bar{x}\pm s$, years)	62.34 ± 15.67	60.94 ± 13.67	0.316	0.754

Gender (*n*)			0.820	0.183
Male	12	9		
Female	10	13		

KPS scores			0.702	0.873
70	3	4		
80	6	6		
90	8	9		
100	5	3		

Evaluable disease sites			0.611	0.434
1	3	5		
≥2	19	17		

Histology (*n*)			0.140	0.709
Squamous	4	5		
Nonsquamous	18	17		

Brain metastasis (*n*)			0.518	0.472
Yes	4	6		
No	18	16		

Stage (*n*)			0.393	0.531
III	7	9		
IV	15	13		

ECOG score (*n*)			0.367	0.545
1	9	11		
2	13	11		

**Table 2:** Clinical efficacy.

	Control group (*n* = 50)	Study group (*n* = 50)
CR, *n* (%)	2 (9.09)	6 (27.27)
PR, *n* (%)	4 (18.18)	7 (31.82)
SD, *n* (%)	10 (45.45)	7 (31.82)
PD, *n* (%)	4 (18.18)	2 (9.09)
ORR, *n* (%)	6 (27.27)	13 (59.09)

*χ*2	4.539	
*P*	0.033	

**Table 3:** Comparison of PFS.

	Events	Median	0.95 LCL	0.95 UCL
Control group (*n* = 22)	16	21.5	14.1	NA
Study group (*n* = 22)	11	28.1	24.5	NA
HR (95% CI)	1.875 (0.877–4.011)			
*P*	0.104			

**Table 4:** Comparison of OS (months).

	Events	Median	0.95 LCL	0.95 UCL
Control group (*n* = 22)	14	27.1	20.7	NA
Study group (*n* = 22)	7	NA	NA	NA
HR (95% CI)	2.56 (1.081–6.06)			
*P*	0.035			

**Table 5:** Comparison of adverse events.

	All grade	Grade ≥3
Control group (*n* = 50)	18	7
Study group (*n* = 50)	20	12
*χ*2	0.772	2.316
*P*	0.380	0.128

## Data Availability

The datasets used during the present study are available from the corresponding author upon reasonable request.
